# Clinical outcome of renin-angiotensin-aldosterone system blockers in treatment of hypertensive patients with COVID-19: a systematic review and meta-analysis

**DOI:** 10.1186/s43044-021-00135-y

**Published:** 2021-02-05

**Authors:** Andrea Laurentius, Brian Mendel, Radityo Prakoso

**Affiliations:** 1 Faculty of Medicine, Universitas Indonesia, Jakarta, Indonesia; 2 Pediatric Cardiology and Congenital Heart Defect Division, National Cardiovascular Center Harapan Kita, Jakarta, Indonesia

**Keywords:** Angiotensin-converting enzyme inhibitors, Angiotensin receptor blockers, COVID-19, Hypertension, Outcome

## Abstract

**Background:**

Novel coronavirus disease 2019 has been stated as global disease pandemic due to its rapid spread worldwide. Up to 30% of coronavirus disease 2019 patients with hypertension are more susceptible to death. Angiotensin-converting enzyme inhibitors and angiotensin receptor blockers have been used as primary line of medication for hypertension; nonetheless, conflicting data arises as numerous studies showed contradictory results.

**Main body:**

Aiming to show clinical outcome of renin-angiotensin-aldosterone system blockers in hospital treatment of hypertensive patients with coronavirus disease 2019, systematically searched literatures through five databases were intensively appraised using *The Grading of Recommendations Assessment*, *Development and Evaluation* checklists for cohort studies. Based on the result evaluation from retrospective cohorts involving more than 15,000 patients across Asia and other regions of the world, ten encompassed studies divided into two subgroups in this meta-review showed that in-hospital hypertensive coronavirus disease 2019 patients receiving antihypertensive drugs were associated with overall risk reduction in subgroup 1 (hazard ratio, HR = 0.96, 95% CI = 0.82–1.12) to no outcome association of all-cause mortalities in subgroup 2 (HR = 0.26, 95% CI = 0.19–0.34). All appraised studies in synergism showed that mortality outcomes were not augmented with the employment of either ACE inhibitor or ARB in subjects.

**Conclusion:**

Therefore, the results support recommendation by the American Heart Association not to discontinue angiotensin-converting enzyme inhibitor or angiotensin receptor blocker regimens in coronavirus disease 2019 patients with hypertension.

**Supplementary Information:**

The online version contains supplementary material available at 10.1186/s43044-021-00135-y.

## Background

Since March 2020, COVID-19 (coronavirus infectious disease–2019) has been declared as a global pandemic by the WHO (World Health Organization), leading death toll up to 1.18 million people worldwide. Due to its envelope properties, severe acute respiratory syndrome–coronavirus–type 2 (SARS-COV-2) could tolerate a wide range of environmental challenges, becoming one of the most infectious agents following HIV (human immunodeficiency virus) and influenza virus. Correspondingly, rapid spread through droplets and fomites by asymptomatic carriers are responsible for its towering transmission rate. Majority of the infected patients were diagnosed after discovering pneumonia as its clinical findings during hospitalization, including 15% with severe comorbidities [1].

Preexisting hypertension represented 30% of comorbidities in COVID-19 patients, who appeared to be more susceptible to death [2]. Angiotensin-converting enzyme inhibitor (ACEi) and angiotensin receptor blockers (ARBs) are primary line of medications for management of high blood pressure through inhibitory effect towards renin-angiotensin-aldosterone system (RAAS) [3]. Furthermore, SARS-COV-2 has been known to infect alveolar epithelia through initial membrane activation of angiotensin converting enzyme 2 (ACE2). Activation of the protein facilitates internalization of viral genetic materials, thereby hijacking its host to self-replicate the viruses [4, 5]. Researches suggested that treatment of hypertension with either ACEi or ARBs enhances the expression of ACE2, which confer predisposition to more severe inflammatory reaction during COVID-19 infection [6]. On the other hand, other reviews stated that both ACEi and ARBs serve protective role against SARS-COV-2 infection by competitive binding to ACE2 protein [7]. Because of inadequate clinical data supporting actual effects of ACEi/ARBs towards prognosis of hypertensive patients infected with COVID-19, optimal strategy for antihypertensive treatments in those patients remains to be elucidated. Controversies on novel uses of RAAS inhibitors have been raised. Therefore, the objective of this systematic review and meta-analysis was to exhaustively determine the linkage between in-hospital usage of ACEi/ARBs and all-cause mortality outcomes among COVID-19 patients with preexisting hypertension.

## Main text

### Search strategy

**Fig. 1:**
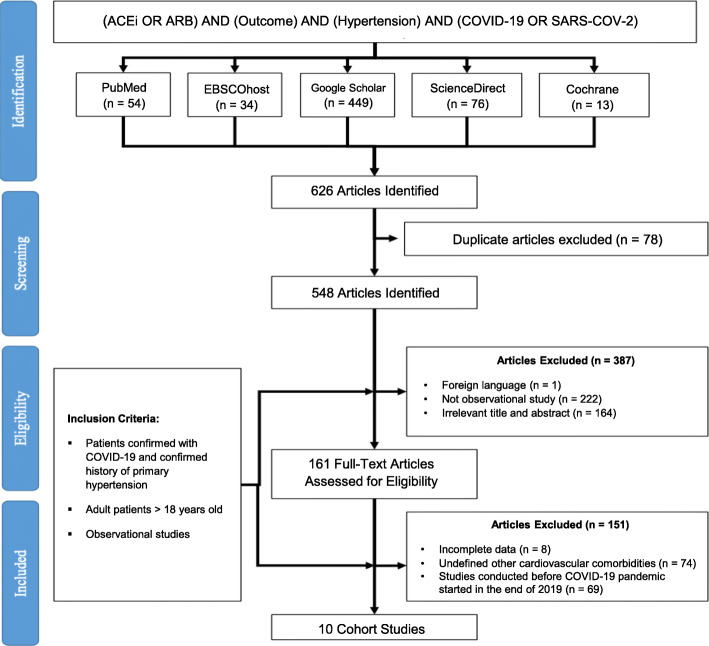
Schematic search strategy results in the standardized PRISMA flow diagram

As this article was subsequently categorized as a systematic review of cohort studies, the search was thoroughly conducted on May 2020 via accessible five medical journal databases, including PubMed, EBSCOhost MEDLINE, Google Scholar, ScienceDirect, and Cochrane. To obtain systematically objective results, three authors performed searching onto different databases using mentioned keywords. Pre-searching protocol of each database was carefully studied to obtain optimal search results based on keywords and title relevancy. The utilized keywords for engine searching are “ACE inhibitors”, “Angiotensin Receptor Blockers”, “Outcome”, “COVID-19”, “SARS-COV-2”, and “Hypertension”. Following appearance of search lists, authors then used program-based filter in each database to rapidly exclude unnecessary studies which do not comply with eligibility criteria. Search strategy results were summarized in the following standardized PRISMA (Preferred Reporting Items for Systematic Review and Meta-Analysis) flow diagrams (Fig. 1).

### Eligibility criteria

All screened articles were then assessed for eligibilities, involving title and abstract skimming, double-checking for full-text availability, and final selecting based on inclusion-exclusion criteria. Studies involving adult patients who had preexisting hypertension and diagnostically confirmed with COVID-19 infection were included in the appraisals. Assessment of inclusion criteria in the screened articles have to consider the following definitions. Preexisting hypertension is defined as history of systolic or diastolic blood pressure of 140 mmHg or 90 mmHg respectively or greater and previous records of antihypertensive medications. According to the American Academy of Pediatrics, patients 18 years old and older are considered as adults [8]. Moreover, COVID-19 was diagnosed with reverse transcription-polymerase chain reaction (RT-PCR) confirmation of nasopharyngeal swab samples and meeting one or more criteria of computerized tomography (CT) chest manifestations based on New Coronavirus Pneumonia Prevention and Control Program and the WHO interim guidance [9, 10]. Included studies were also either prospective or retrospective cohorts comprising of antihypertensive treatment group with ACEi/ARB and non-ACEi/ARB during follow-up observation in hospitals. Studies that involve hypertensive patients with undefined cardiovascular comorbidities were excluded, as well as studies prior to the time when COVID-19 pandemic hit in the end of 2019.

### Critical appraisal

The eligible studies were intensively appraised using GRADE (Grading of Recommendations Assessment, Development and Evaluation) checklists for systematic review and meta-analysis of pooled cohort studies [11]. It comprises of five main points depicting completion of checklists required for each section to assess article’s limitations, inconsistency, indirectness, imprecision, and publication bias as interim analysis. Assessment based on GRADE criteria also involves valuing importance of the articles based on study data collection, further refining final qualification of each study. The critical appraisal of each study was conducted based on unanimous consensus from all authors discussing on this systematic review. Level of completeness in each checklist is qualitatively shown in Table 1.

**Table 1:** GRADE evidence profile of selected studies [12–21]

**Summary of findings**												**Quality assessment**	
**Study (year)**	**Settings**	**Study design**	**Length of study**	**Sample size**		**Grading of Hypertension**	**Duration of follow-up**	**Secondary outcomes (comorbidities)**	**Primary outcomes (all-cause mortality)**		**Hazard ratio (95% CI)**	**Checklists**	**Results**
**Lee et al. (2020)** [13]	Centers for Disease Control and Prevention (Korea)	Retrospective cohort	Jan 2020 until Mar 2020	*N* = 8266		Minimal grade 1	2 months	Diabetes mellitus, cancer, COPD, stroke, coronary artery disease, heart failure, chronic renal disease	*N* = 112		1.07 (0.68–1.68)	**Limitations**	Not serious
				**Non-ACEi/ARB,** 977	**ACEi/ARB,** 7289				**Non-ACEi/ ARB,** 62	**ACEi/ ARB,** 50		**Inconsistency**	Serious
												**Indirectness**	Not serious
												**Imprecision**	Serious
												**Publication Bias**	Undetected
												**Quality**	**High**
**Li et al. (2020)** [12]	Central Hospital of Wuhan (China)	Retrospective cohort	15 Jan 2020 until 15 Mar 2020	*N* = 362		Minimal grade 1	2 months	Cerebrovascular disease, cardiovascular disease, diabetes, chronic kidney disease	*N* = 77		0.76 (0.44–1.33)	**Limitations**	Not serious
				**Non-ACEi/ARB,** 247	**ACEi/ARB,** 115				**Non-ACEi/ ARB,** 56	**ACEi/ ARB**, 21		**Inconsistency**	Not serious
												**Indirectness**	Not serious
												**Imprecision**	Not serious
												**Publication Bias**	Undetected
												**Quality**	**High**
**Mehra et al. (2020)** [14]	Multihospital International Registry (Asia, Europe, and North America)	Retrospective cohort	20 Dec 2019 until 15 Mar 2020	*N* = 2346		Minimal grade 1	n/a	Heart disease, diabetes mellitus, COPD	N = 184		0.24 (0.17–0.33)	**Limitations**	Serious
				**Non-ACEi/ARB,** 920	**ACEi/ARB,** 1426				**Non-ACEi/ ARB,** 130	**ACEi/ ARB,** 54		**Inconsistency**	Not serious
												**Indirectness**	Not serious
												**Imprecision**	Not serious
												**Publication bias**	Undetected
												**Quality**	**High**
**Meng et al. (2020)** [17]	Shenzhen Third People’s Hospital (China)	Retrospective cohort	11 Jan 2020 until 23 Feb 2020	*N* = 42		Grade 2 or grade 3 hypertension according to ESC	n/a	Type 2 diabetes or coronary heart disease	*N* = 1		0.46 (0.018–12.14)	**Limitations**	Serious
				**Non-ACEi/ARB,** 25	**ACEi/ARB,** 17				**Non-ACEi/ ARB,** 1	**ACEi/ ARB,** 0		**Inconsistency**	Serious
												**Indirectness**	Not serious
												**Imprecision**	Not serious
												**Publication bias**	Detected
												**Quality**	**Low**
**Yang et al. (2020)** [20]	Hubei Provincial Hospital of Traditional Chinese Medicine (China)	Retrospective cohort	1 Nov 2019 until 31 Dec 2019	*N* = 126		Minimal grade 1	3 months	Diabetes or cardiopathy	*N* = 13		0.35 (0.08–1.51)	**Limitations**	Not serious
				**Non-ACEi/ARB,** 83	**ACEi/ARB,** 43				**Non-ACEi/ARB,** 11	**ACEi/ARB,** 2		**Inconsistency**	Not serious
												**Indirectness**	Not serious
												**Imprecision**	Serious
												**Publication bias**	Undetected
												**Quality**	**Moderate**
**Zhou et al.** [18] **(2020)**	Wuhan Fourth Hospital, Hubei Province (China)	Retrospective cohort	25 Jan 2020 until 20 Feb 2020	36		Minimal grade 1	25 days	Diabetes, cerebrovascular disease, cardiovascular disease, COPD, chronic kidney disease, malignancy, rheumatoid arthritis	*N* = 7		0.14 (0.009–2.2)	**Limitations**	Serious
				**Non-ACEi/ARB,** 21	**ACEi/ARB,** 15				**Non-ACEi/ARB,** 5	**ACEi/ARB,** 2		**Inconsistency**	Serious
												**Indirectness**	Not serious
												**Imprecision**	Very serious
												**Publication bias**	Detected
												**Quality**	**Low**
**Fosbol et al. (2020)** [16]	Danish national administrative registry (Denmark)	Retrospective cohort	22 Feb 2020 until 4 May 2020	*N* = 4480		Minimal grade 1	End of study period (4 May 2020)	Diabetes, myocardial infarction, cancer, cerebrovascular diseases, COPD, chronic kidney diseases	*N* = 197		0.94 (0.65–1.37)	**Limitations**	Not serious
				**Non-ACEi/ARB,** 3585	**ACEi/ARB,** 895				**Non-ACEi/ARB,** 36	**ACEi/ARB,** 161		**Inconsistency**	Serious
												**Indirectness**	Not serious
												**Imprecision**	Not serious
												**Publication bias**	Undetected
												**Quality**	**High**
**Pan et al. (2020)** [21]	Renmin Hospital of Wuhan University (China)	Retrospective single-center study	4 Jan 2020 until 14 Feb 2020	*N* = 282		Minimal grade 1	30 days	Diabetes, coronary heart diseases, cerebrovascular diseases, chronic liver disease, chronic kidney disease	*N* = 67		0.31 (0.1–0.89)	**Limitations**	Not serious
				**Non-ACEi/ARB,** 241	**ACEi/ARB,** 41				**Non-ACEi/ARB,** 63	**ACEi/ARB,** 4		**Inconsistency**	Serious
												**Indirectness**	Not serious
												**Imprecision**	Serious
												**Publication bias**	Undetected
												**Quality**	**Moderate**
**Reynolds et al. (2020)** [15]	New York University Langone Health (New York)	Retrospective cohort	1 Mar 2020 until 15 Apr 2020	*N* = 2005		Minimal grade 1	40 days	Heart failure, myocardial infarction, diabetes, chronic kidney disease, obstructive lung disease	*N* = 501		0.97 (0.79–1.19)	**Limitations**	Not serious
				**Non-ACEi/ARB,** 986	**ACEi/ARB,** 1019				**Non-ACEi/ARB,** 249	**ACEi/ARB,** 252		**Inconsistency**	Serious
												**Indirectness**	Not serious
												**Imprecision**	Not serious
												**Publication bias**	Undetected
												**Quality**	**High**
**Zhang et al (2020)** [19]	Hubei’s Nine Hospital (China)	Retrospective cohort	31 Dec 2019 until 7 Mar 2020	*N* = 1128		Minimal grade 1	1 month	Diabetes, cardiac, or renal diseases	*N* = 99		0.34 (0.14–0.83)	**Limitations**	Not serious
				**Non-ACEi/ARB,** 348	**ACEi/ARB,** 174				**Non-ACEi/ARB,** 92	**ACEi/ARB,** 7		**Inconsistency**	Serious
												**Indirectness**	Not serious
												**Imprecision**	Serious
												**Publication Bias**	Undetected
												**Quality**	**Moderate**

### Data extraction

Data extraction from each selected article included authorships, study year and design, population characteristics, population size, duration of follow-up, defined comorbidities, hypertension grading, duration of follow-up, and outcome. The data extraction was conducted alongside with assessing study’s qualities in terms of GRADE checklists. The primary endpoint of this review is defined as the all-cause mortality outcome of hypertensive patients with COVID-19; nevertheless, the secondary endpoints were comorbidities in hospitalized hypertensive patients due to COVID-19.

### Statistical meta-analysis

Extracted values of primary endpoint were transposed into hazard ratio (HR) via generic inverse variance. Adjusted HR was calculated via logistic hazard ratio through computational random effects model. Estimation of 95% confidence interval (CI) and *p* value was added to support risk evaluation of the endpoint. To optimize metadata summary from different articles, interstudy forest and funnel plot comparison was conducted via the Review Manager v.5.4 and Microsoft Office Excel software. Additionally, chi-square tests and Cochrane Q score were used to quantitatively examine heterogeneity between studies. Two-tailed *p* value of less than 0.05 is considered statistically significant [11].

### Search results

A comprehensive search was accomplished using five different online databases. Of all 626 potential articles confined to combination of written keywords and Boolean operators, 78 were found to be duplicate articles. The duplicated articles were manually identified using the EndNote X9 software. Hence, the remaining 548 were screened based on both title and abstract relevancies with up to more than 380 articles were omitted from selected references. Thus, 161 full texts of the remaining articles were further assessed for eligibility criteria. After blind review by each author, a number of 151 articles were excluded due to violation of agreed inclusion criteria, such as incomplete data or undefined other cardiovascular comorbidities. Ten articles were included into the finally selected articles of the review. Description of these sequential steps in systematic study selection is done through constructing the PRISMA diagram, as shown in Fig. 1 [22].

### Studies appraisal

Critical appraisal was extensively conducted on ten observational studies via GRADE checklists [11]. Based on the appraisal checklists, five out of ten studies [12–16] are considered to be high quality, due to their clear and concise methodological process. Furthermore, their high sample size (*n* > 300) involvement signifies low risk of imprecision. When sample size proportionally correlated with the difficulty of conducting the study, it turns out that well-controlled bias and adjustment of end results could minimize study limitations focusing on result importance and validity [12–16]. Li et al. turns out to have the highest quality assessment which is characterized by no serious bias in all points [12]. Moreover, risk of serious limitation and inconsistency bias due to unclear methodological descriptions are found in Meng et al. studies, followed by Zhou et al. Two publication bias are, however, detected in Zhou et al. and Meng et al. as their studies are scattered asymmetrically in the funnel plot as outliers against pooled small studies [17, 18]. The other three studies exhibit moderately qualified appraisal for one to two serious bias in the points, as well as medium amount of their study samples [19–21]. The complete results are qualitatively shown in the following table, shown in Table 1.

### Study results

#### Comparative data extraction

Based on the result evaluation from ten retrospective cohorts involving more than 15,000 patients across Asia and other regions of the world, majority of the studies were conducted to assess the probable outcomes of RAAS blockers within hypertensive patients infected with COVID-19. Thus, most of the pooled studies were recently held during early outbreak of COVID-19. Data collection of patients’ medical records from respective hospitals mostly began in January 2020; nevertheless, Yang et al.’s study yields the earliest time of record collection, that was on November 2019 in the city of Wuhan [20]. Additionally, Meng et al. defined hypertension based on its grading in patients infected with COVID-19 to observe any substantial differences in mortality outcomes [17]. Largest sample size was seen in study done by Lee et al., followed by Fosbol et al. and Mehra et al. [13, 14, 16]. Smallest samples included in the studies are Zhou et al., followed by Meng et al. [17, 18]. Propensity matching of total included samples in the studies failed to show 1:1 size ratio between ACEi/ARB group and non-ACEi/ARB group. Furthermore, selection of study samples in the studies were based on existence of preexisting hypertension prior to COVID-19 pandemic, thus exhibiting required criteria in the sample populations. Duration of follow-up in all studies ranged between 1 and 3 months, which took time mostly on January till March 2020. This period of time was believed as representation of worldwide exponential spreading of COVID-19. Location of data extraction majorly took place in central hospitals or university hospitals, with the exception in the study done by Mehra et al., which utilized multihospital registry across nations [14]. Table 1 summarizes data extractions from the selected observational studies.

#### Primary and secondary outcomes

Patient characteristics between group of ACEi/ARB and non-ACEi/ARB represent distribution of potential confounding factors. Significant difference of mean age between groups was found in Lee et al. and Zhou et al.’s study as other studies exhibit similar age distribution between groups, except unknown *p* value of studies by Mehra et al., Fosbol et al., and Reynolds et al. [13–16, 18]. Discrepancy of female-to-total patient ratio between groups suggests risk of bias of primary outcome in Lee et al.’s study, for each gender possesses contrasting effects of ACEi/ARB toward cardiovascular outcome [13]. Besides, seven studies obtained predominant data sources in Asia and two of which in Europe, except the multinational cohort study done by Mehra et al. The consistency of race belonging to the ten studies varied due to the fact that the studies’ coverage was limited to single country inhabited with or without immigrants, except for Mehra et al. The overall primary outcome of this review is all-cause mortality of pre-existing hypertensive patients infected with COVID-19; on the other hand, the secondary outcomes would be to evaluate comorbidities in patients that potentially adjust the survival value of ACEi/ARB in treating hypertension. All defined comorbidities were generalized into five main subgroups as they represented the most frequent cause of deaths in COVID-19 patients, comprising of diabetes mellitus, coronary artery disease, congestive heart failure, chronic kidney disease or renal failure, and cerebrovascular disease or stroke. Study conducted by Lee et al. and Meng et al. exhibited disparities of comorbidities between observed groups, which is statistically proven by respective *p* value under 0.05 [13, 17]. This may generate unimportant research results due to its nature of high-risk bias among populations in the groups. Table 2 qualitatively summarizes the data of patient characteristics in all selected studies.

**Table 2:** Summarized data of patients’ characteristics [12–21]

**Characteristics**	**Lee et al. (2020) [13]**			**Li et al. (2020) [12]**			**Mehra et al. (2020) [14]**			**Meng et al. (2020) [17]**			**Yang et al. (2020) [20]**		
	ACEi/ ARB	Non-ACEi/ ARB	*P value*	ACEi/ ARB	Non-ACEi/ ARB	*P value*	ACEi/ ARB	Non-ACEi/ ARB	*P value*	ACEi/ ARB	Non-ACEi/ ARB	*P value*	ACEi/ARB	Non-ACEi/ ARB	*P value*
	(*n* = 977)	(*n* = 7289)		(*n* = 115)	(*n* = 247)		(*n* = 1426)	(*n* = 920)		(*n* = 17)	(*n* = 25)		(*n* = 43)	(*n* = 83)	
Median Age^i^ (Range)	64	42	< 0.001*	65	67	0.22	49		n/a	64	65	0.91	65	67	0.122
	(52–77)	(23–60)		(57–73)	(60–75)		(38–60)			(55–69)	(55–68)		(57–72)	(62–75)	
Gender, female (%)	56.3	62.2	0.002*	40.9	51	0.56	40		n/a	47.1	40	0.9	50.6	51.2	0.952
Race, Asian (%)	100	100	> 0.99	100	100	> 0.99	19.3		n/a	100	100	> 0.99	100	100	> 0.99
Comorbidities															
Diabetes (%)	97.3	8.55	< 0.001*	36.5	34.4	0.7	14.3		n/a	5.8	20	< 0.001*	13	25	0.99
CAD (%)	19.65	3.81	< 0.001*	23.5	14.2	0.03*	11.34		n/a	n/a	n/a	n/a	16.3	19.3	0.68
Heart Failure (%)	4.2	0.59	< 0.001*	4.3	2	0.36	2.12		n/a	5.8	28	< 0.001*			
CKD (%)	4.2	0.59	< 0.001*	11.3	8.9	0.47	n/a		n/a	n/a	n/a	n/a	0	3.6	0.207
Stroke (%)	12.3	1.59	< 0.001*	23.5	16.6	0.12	n/a		n/a	n/a	n/a	n/a	9.3	7.2	0.683
All-Cause Mortality (n)	50	62	0.81	21	56	0.34	54	130	< 0.001*	0	1	0.647	2	11	0.216
Hazard Ratio^ii^ (95% CI)	1.07 (0.68–1.68)			0.76 (0.44–1.33)			0.24 (0.17–0.33)			0.46 (0.018–12.14)			0.35 (0.08–1.51)		
**Characteristics**	**Zhang et al. (2020) [19]**			**Fosbol et al. (2020) [16]**			**Pan et al. (2020) [21]**			**Reynolds et al. (2020) [15]**			**Zhou et al. (2020) [18]**		
	ACEi/ ARB	Non-ACEi/ ARB	*P value*	ACEi/ ARB	Non-ACEi/ ARB	*P value*	ACEi/ ARB	Non-ACEi/ ARB	*P value*	ACEi/ ARB	Non-ACEi/ ARB	*P value*	ACEi/ARB	Non-ACEi/ ARB	*P value*
	(*n* = 174)	(*n* = 348)		(*n* = 895)	(*n* = 3385)		(*n* = 41)	(*n* = 241)		(*n* = 1019)	(*n* = 986)		(*n* = 15)	(*n* = 21)	
Median Age^i^ (Range)	64	64	> 0.2	73	50	n/a	70	69	0.889	64		n/a	58	69	0.001*
	(56–68)	(56–69)		(61–81)	(37–64)		(63–76)	(62–76)		(54–75)			(48–68)	(62–76)	
Gender, female (%)	46	43.4	0.052	44.9	53.9	n/a	61	57.3	0.105	49.2		n/a	40	52.4	0.516
Race, Asian (%)	100	100	> 0.99	< 12.5	< 15.2	n/a	100	100	> 0.99	7.5		n/a	100	100	> 0.99
Comorbidities															
Diabetes (%)	23	24.7	> 0.2	24.2	5.4	n/a	< 41.5	< 51	0.499	39.7		n/a	25		n/a
CAD (%)	13.8	13.2	> 0.2	21.6	5.2	n/a				10.6		n/a	19.4		n/a
Heart Failure (%)	n/a	n/a	n/a	14.6	3.1	n/a				16.1		n/a			
CKD (%)	4	3.2	> 0.2	7.5	2.9	n/a				25		n/a	n/a		n/a
Stroke (%)	2.3	2.3	> 0.99	19.4	6.4	n/a				n/a		n/a	n/a		n/a
All-Cause Mortality (n)	7	92	0.02*	161	36	0.83	4	63	0.037*	252	249	0.78	2	5	0.162
Hazard Ratio^ii^ (95% CI)	0.34 (0.14–0.83)			0.94 (0.65–1.37)			0.31 (0.1–0.89)			0.97 (0.79–1.19)			0.14 (0.009–2.2)		

^i^Age is calculated as unit of year. Asterisked *p* values (*) indicate statistically significant data. Mean difference of age is calculated via independent *T* test; other characteristics are calculated via Chi-square test

^ii^Hazard ratio < 1 shows risk reduction of all-cause mortality in the ACEi/ARB-treated group

#### Meta-analysis

**Fig. 2:**
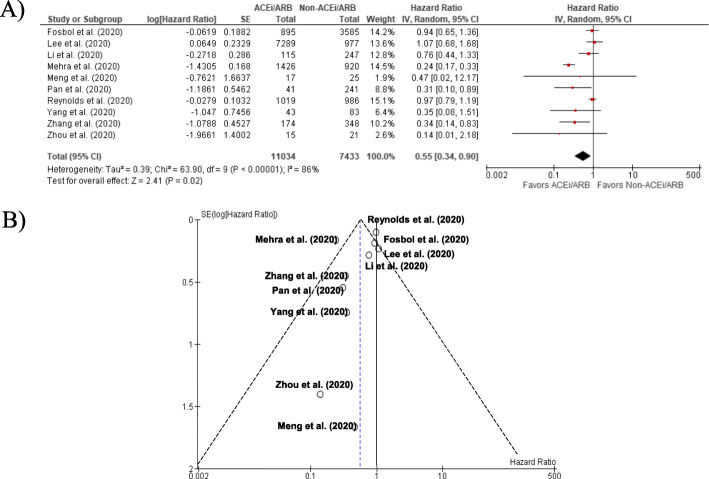
**a** Random effects model of pooled hazard ratio comparison: outcome of ACEi/ARB. **b** Overall estimate of study publication bias scattered in funnel plot

**Fig. 3:**
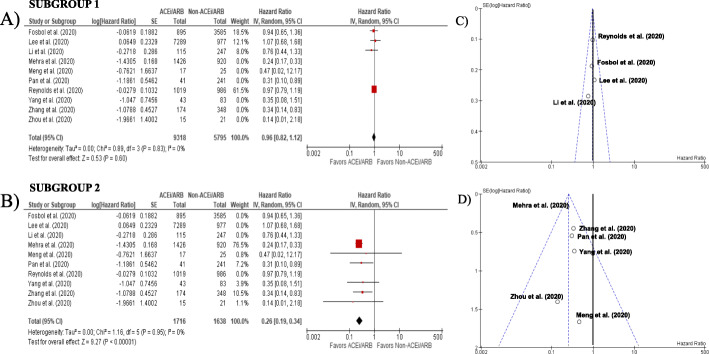
Additional adjusted meta-analysis of studies in subgroup 1 **a** and subgroup 2 **b**. Estimate of subgroup 1 **c** and subgroup 2 **d** publication bias scattered in funnel plot

Few detailed studies in the literature concerns the usage of ACEi/ARB that could exacerbate severity of COVID-19 in hypertensive patients. Profound pooled analysis of multiple international studies could provide insights of ACEi/ARB clinical effects in hypertensive COVID-19 patients, therefore, further clarifying emerging information regarding ACEi/ARB severe prognostication in COVID-19 patients via description of its pharmacological pathophysiology. Random effects meta-analysis model of ten studies suggested significant heterogeneities, proven by high yield of heterogeneity index (*I*^2^ = 86%) and statistically significant Cochrane Q test (*p* = < 0.00001/df = 9), shown in Fig. 2. Choosing random effects model in this pooled study was to assist in minimizing unobserved heterogeneity which is not correlated with independent variables, and thus yielding proportional and conservative quantification of each study result. As tipping point of analysis to assess potential effect of unmeasured confounder, depiction of study inter-relation into funnel plot was divided into two major subgroups, as shown in Fig. 3. Both subgroups exhibit zero heterogeneity indexes and statistically insignificant Cochrane Q test, denoting moderately homogeneous study results. The *p* value of overall effects was 0.53 (*z* score = 0.6) in subgroup 1, which was different from that of subgroup 2, supporting differences in significancy of each pooled subgroup towards clinical application. The overall effect of subgroup 2 (HR = 0.26, 95% CI = 0.19–0.34) exerts more favorable effect of ACEi/ARB than that of subgroup 1 (hazard ratio, HR = 0.96, 95% CI = 0.82–1.12) in reducing all-cause mortalities of hypertensive COVID-19 patients. Nevertheless, the result indicates confirmed usage of ACEi/ARB associated with reduced to nil-effect outcome mortality in adult hypertensive COVID-19 patients.

## Discussion

Renin-angiotensin-aldosterone system (RAAS) has been reported to play a central role in regulating hypertension and acute lung injury. Therefore, effective therapeutic strategy targeting RAAS, such as ACEi and ARBs, through inhibition of ACE/AngII/AT1R axis is the commonest used drugs for hypertension. Recent meta-analysis study on COVID-19 comorbidities identified in patients were hypertension (15.8%), cardiocerebrovascular diseases (11.7%), diabetes (9.4%), respiratory illness (1.4%), and renal diseases (0.8%) [23]. Along with original data from Wuhan city, it showed 10.5% mortality among person with COVID-19 who also had comorbidities. Due to the fact that comorbidities found in participants of selected studies may predispose bias, focusing on extracting adjusted data would be important [24]. Hypertensive COVID-19 patients are prone to progress into grave cases, and choices should be made to determine the sole effect of RAAS blockers on COVID-19 patients with pre-existing hypertension.

According to a special report by Vaduganathan et al., ACE2 is expressed in multiple organs, including the heart, kidneys, and lung alveolar cells, which are the target of severe acute respiratory syndrome–coronavirus–type 2 (SARS-COV-2) regarding its function as binding sites for the virus [25]. This fact raised concerns regarding the utilization of ACE inhibitors and ARBs which may exacerbate severity of COVID-19 patients. ACE inhibitors and ARBs possess heterogeneous effects on ACE2 in animal studies, while little data exist on the pharmacokinetic effect of ACE inhibitors and ARBs on human. Many researches had conflicting data, indicating various effects on ACE2 across different molecules of ACE inhibitors and ARBs.

In response to the conflicting data, the American Heart Association (AHA) has recommended new guidelines for management of hypertension in patients visiting healthcare during COVID-19. The recommendation suggests hypertensive COVID-19 patients should not stop taking either ACEi or ARB [26]. Controversies arose when it was brought to light that SARS-COV-2 could bind to ACE2 receptor to acquire access into cells. Several animal studies indicate that ACEi and ARB increase expression of ACE2 and raised wide concerns that using these types of antihypertensives exacerbate susceptibility to the virus. Increased level of ACE2 on one hand may facilitate infection by COVID-19 and elevate the risk of developing severe and fatal COVID-19 manifestations. Reduced expression of ACE2 could trigger pulmonary edema and reduced lung function; in contrast, other studies claimed that it reduces systemic levels of angiotensin-2 which may be protective against lung damages in COVID-19 patients [27].

Furthermore, Tignanelli et al. suggested hypertensive patients have hyperactive RAAS activation through angiotensin-2 which has been assumed to conciliate acute lung injury including lung inflammation, fibrosis, and even edema during SARS-COV-2 virus infection. Activation of ACE2 results in low quantity of angiotensin-2, and its impairment would result in excessive release of angiotensin-2. Angiotensin-2 positively adjusts the expression of cytokines through angiotensin 2 type 1 receptor (AT1R) activation [28]. Correspondingly, study from China showed higher angiotensin-2 serum level in a group of 12 SARS-COV-2-infected patients compared to the uninfected and was synergistically associated with lung damage and viral loads [29]. Not only related with abysmal prognosis of COVID-19 disease, hypertension was also linked to decreased amounts of ACE2 expression. Studies suggested that COVID-19 interaction with ACE2 receptor may cease residual ACE2 activity, elevating angiotensin-2 levels, and several studies stated that binding of ARB to the AT1R may stabilize the AT1R-ACE2 complex and halt SARS-COV-2-ACE2 interface [30]. Currently, all guidelines recommend that patients with hypertension comorbidity should not discontinue ACEi or ARBs in this setting except for clinical reasons.

Correspondingly, this meta-analysis of pooled several studies provide deeper insights of clinical outcome of ACEi/ARB in hypertensive COVID-19 patients, hoping to clarify the recommendation of RAAS-based antihypertensive usage, as well as other conflicting sources. Our appraised studies exhibit high heterogeneity (*I*^2^ = 84%) according to Cochrane Q test of pooled meta-analysis. Drawing conclusions from pooled analysis with high heterogeneity was inappropriate [31]. Therefore, divided grouping on studies was done on the funnel plot basis indicating two separated subgroups (see Fig. 3). Moreover, the proximate causation of these groupings is to observe any significant differences of ACEi/ARB outcome toward all-cause mortality of hypertensive COVID-19 patients between the totally pooled with the subgrouped meta-analysis. Two subgroups (both *I*^2^ of 0%) present such statistically significant differences of outcome results, alongside with evidence of RAAS blockers protective effects in minimizing mortalities of hypertensive COVID-19 patients (see subgroup 2, Fig. 3b). Nevertheless, acknowledging larger sample size in subgroup 1 (*n* = 15,113), with no dramatic outcome association, may question the certainty of pooled evidences in subgroup 2 (*n* = 3354) [31]. This difference is also probably due to study distinctions in which the characteristics of research population were conducted. In the analysis, Mehra et al. and Zhang et al. are the only studies which perspicuously stated hypertension as the sole comorbidity of COVID-19 patients, contributing to lower all-cause mortality outcome during treatment with ACEi/ARB [14, 19]. Furthermore, majority of participants involved in subgroup 2 analysis are predominantly originated from single country in China, which pose discrepancy from subgroup 2 with the exception of study by Li et al. [12]. Li et al. is the only subgroup 2 study conducted in China with the highest involvement of participants among the others. Nonetheless, all appraised studies in synergism showed that mortality outcomes were not augmented with the employment of either ACE inhibitor or ARB in subjects [12–21].

There are several limitations to this conducted study. In view of the fact that majority of the participants in the studies are originated from China or Asian countries, researches from non-Asian countries should also be conducted to secure more generalized and accepted utilization of ACE-inhibitor or ARB in hypertensive COVID-19 patients. Furthermore, several selected studies failed to mention in lucidity regarding the scope of follow-up duration, grade of hypertension, age, gender, and the other standardized baseline characteristics, which may affect outcome results. No additional outcomes other than mortality was thought to be assessed in all selected studies, such as in hospital bedrest duration or specific treatments, as they may highlight differences of hospital’s health service level in minimizing mortality rates.

## Conclusions

All encompassed studies showed that hypertensive patients with COVID-19 who received ACE inhibitor or ARB in hospital treatment were associated to lower risk of all-cause mortalities compared to the non-users. The results support recommendation by the American Heart Association not to discontinue previous treatment of ACEi/ARBs among COVID-19 patients with hypertension. Studies also showed that hospitalized ACEi or ARB patients have better prognosis than untreated hypertensive patients regardless of their COVID-19 infection status. Thus, further researches are required as that majority of the studies are originated from Asian countries along with comprehensive standardization of baseline characteristics.

## Supplementary Information

Additional file 1: **Figure 1.** Schematic search strategy results in the standardized PRISMA flow diagram. **Figure 2.** A) Random effect model of pooled hazard ratio comparison: Outcome of ACEi/ARB. B) Overall estimate of study publication bias scattered in funnel plot. **Figure 3.** Additional adjusted meta-analysis of studies in subgroup 1 (A) and subgroup 2 (B). Estimate of subgroup 1 (C) and subgroup 2 (D) publication bias scattered in funnel plot.

## Data Availability

All data generated or analyzed during this study are included in this published article (and its supplementary information files).

## Abbreviations

ACE: Angiotensin-converting enzyme
ACEi: Angiotensin-converting enzyme inhibitors
AHA: American Heart Association
ARB: Angiotensin receptor blockers
CI: Confidence interval
COVID-19: Coronavirus infectious disease–2019
CT: Computed tomography
Df: Degree of freedom
HIV: Human immunodeficiency virus
HR: Hazard ratio
PRISMA: Preferred Reporting Items for Systematic Review and Meta-analysis
RAAS: Renin-angiotensin-aldosterone system
RT-PCR: Reversed transcriptase-polymerase chain reaction
SARS-COV-2: Severe acute respiratory syndrome–coronavirus–type 2
STROBE: Strengthening the Reporting of Observational Studies in Epidemiology
WHO: World Health Organization
