# Supplementary material for: Clinical outcome of renin-angiotensin-aldosterone system blockers in treatment of hypertensive patients with COVID-19: a systematic review and meta-analysis
Source: Egypt Heart J. 2021 Feb 5;73:13. doi: 10.1186/s43044-021-00135-y (PMC7863036; doi:10.1186/s43044-021-00135-y)
Supplement: Supplementary file 1 — Figure 1. Schematic search strategy results in the standardized PRISMA flow diagram. Figure 2. A) Random effect model of pooled hazard ratio comparison: Outcome of ACEi/ARB. B) Overall estimate of study publication bias scattered in funnel plot. Figure 3. Additional adjusted meta-analysis of studies in subgroup 1 (A) and subgroup 2 (B). Estimate of subgroup 1 (C) and subgroup 2 (D) publication bias scattered in funnel plot. [file 43044_2021_135_MOESM1_ESM.pptx]

## Slide 1
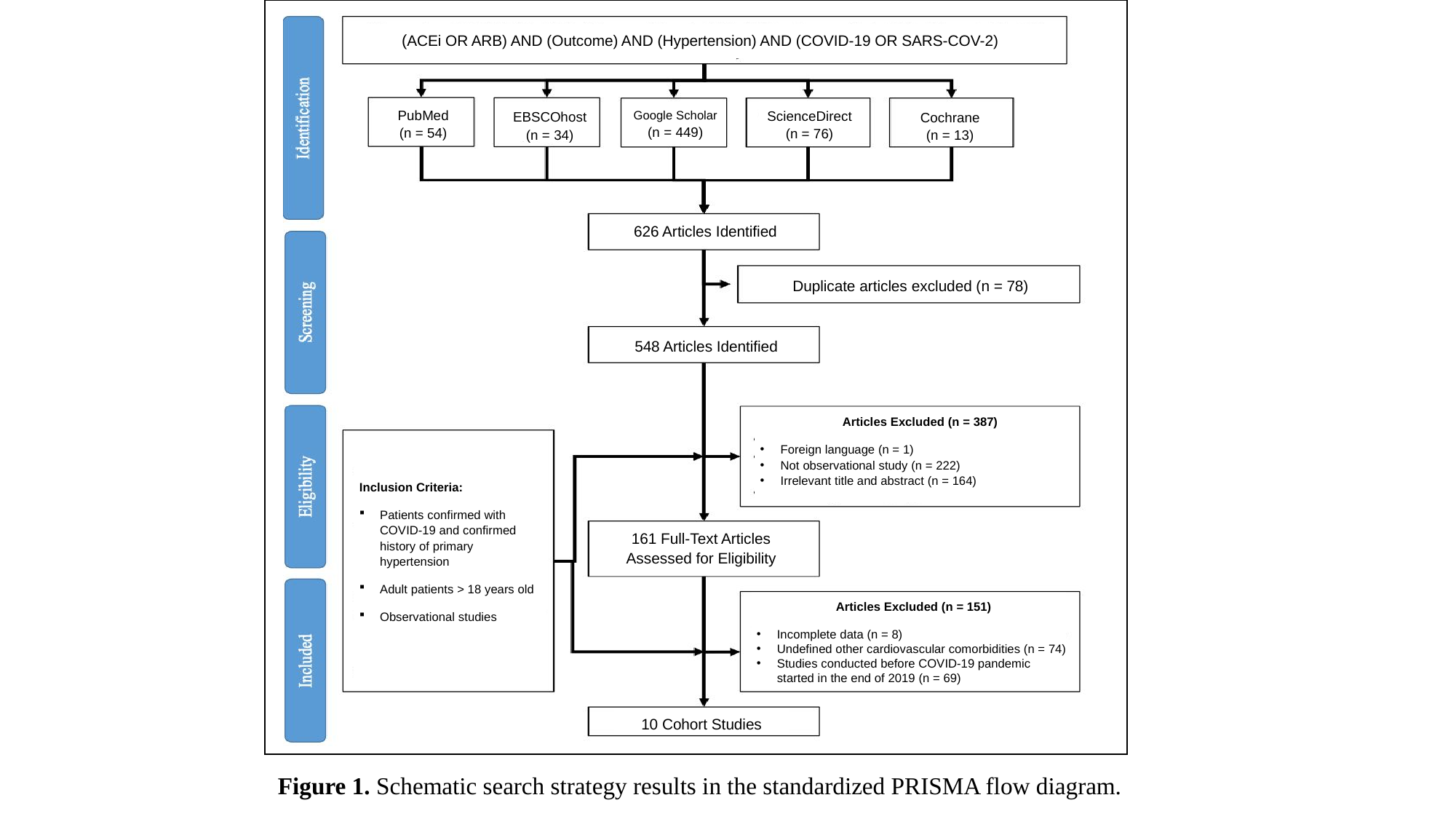

PubMed
(n = 54)
(ACEi OR ARB) AND (Outcome) AND (Hypertension) AND (COVID-19 OR SARS-COV-2)
ScienceDirect
(n = 76)
Google Scholar
(n = 449)
EBSCOhost
(n = 34)
Cochrane
(n = 13)
626 Articles Identified
Duplicate articles excluded (n = 78)
548 Articles Identified
Articles Excluded (n = 387)
Foreign language (n = 1)
Not observational study (n = 222)
Irrelevant title and abstract (n = 164)
Inclusion Criteria:
Patients confirmed with COVID-19 and confirmed history of primary hypertension
Adult patients > 18 years old
Observational studies
161 Full-Text Articles
Assessed for Eligibility
Articles Excluded (n = 151)
Incomplete data (n = 8)
Undefined other cardiovascular comorbidities (n = 74)
Studies conducted before COVID-19 pandemic started in the end of 2019 (n = 69)
10 Cohort Studies
Figure 1. Schematic search strategy results in the standardized PRISMA flow diagram.

## Slide 2
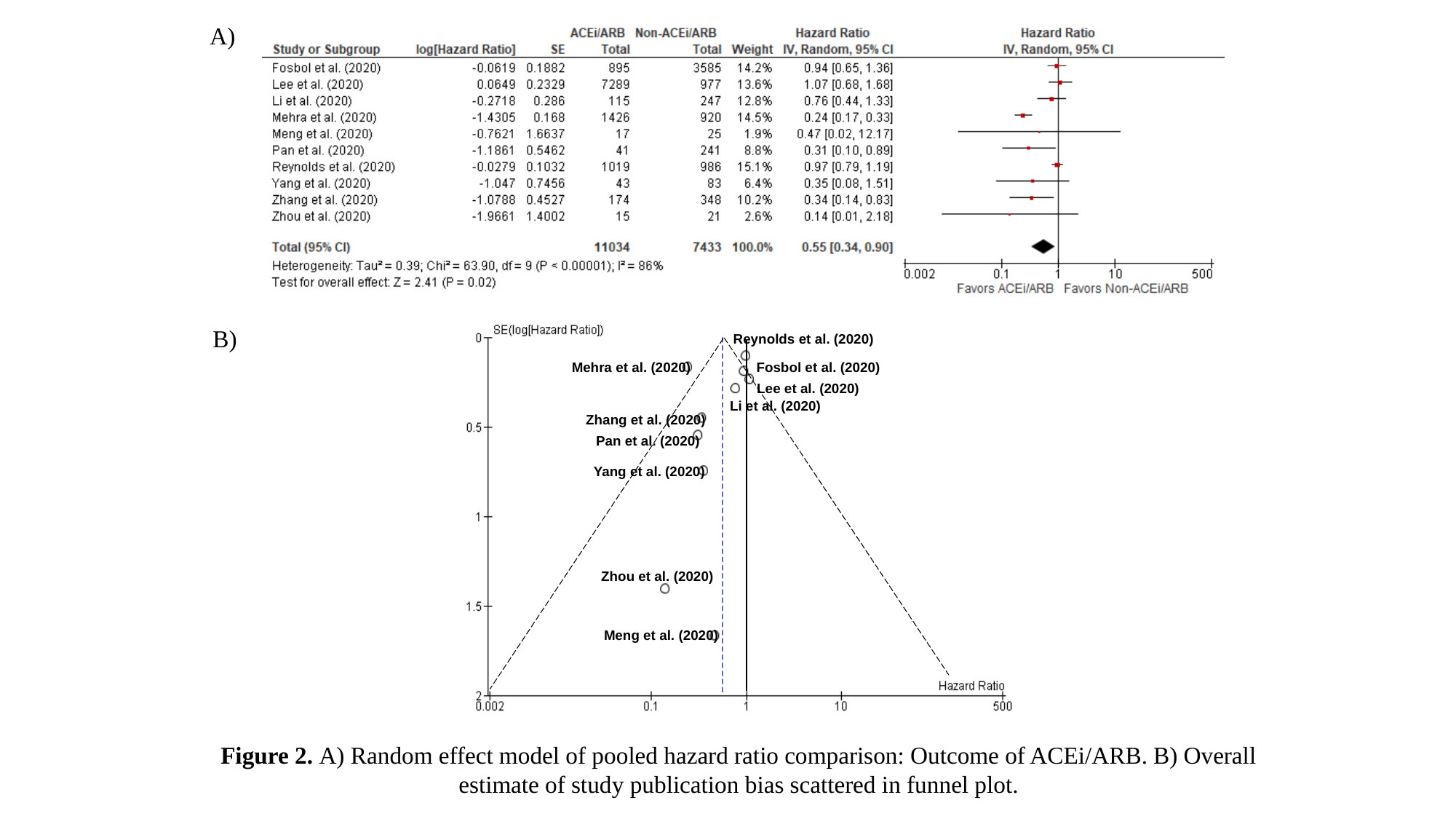

A)
B)
Reynolds et al. (2020)
Mehra et al. (2020)
Fosbol et al. (2020)
Lee et al. (2020)
Li et al. (2020)
Zhang et al. (2020)
Pan et al. (2020)
Yang et al. (2020)
Zhou et al. (2020)
Meng et al. (2020)
Figure 2. A) Random effect model of pooled hazard ratio comparison: Outcome of ACEi/ARB. B) Overall estimate of study publication bias scattered in funnel plot.

## Slide 3
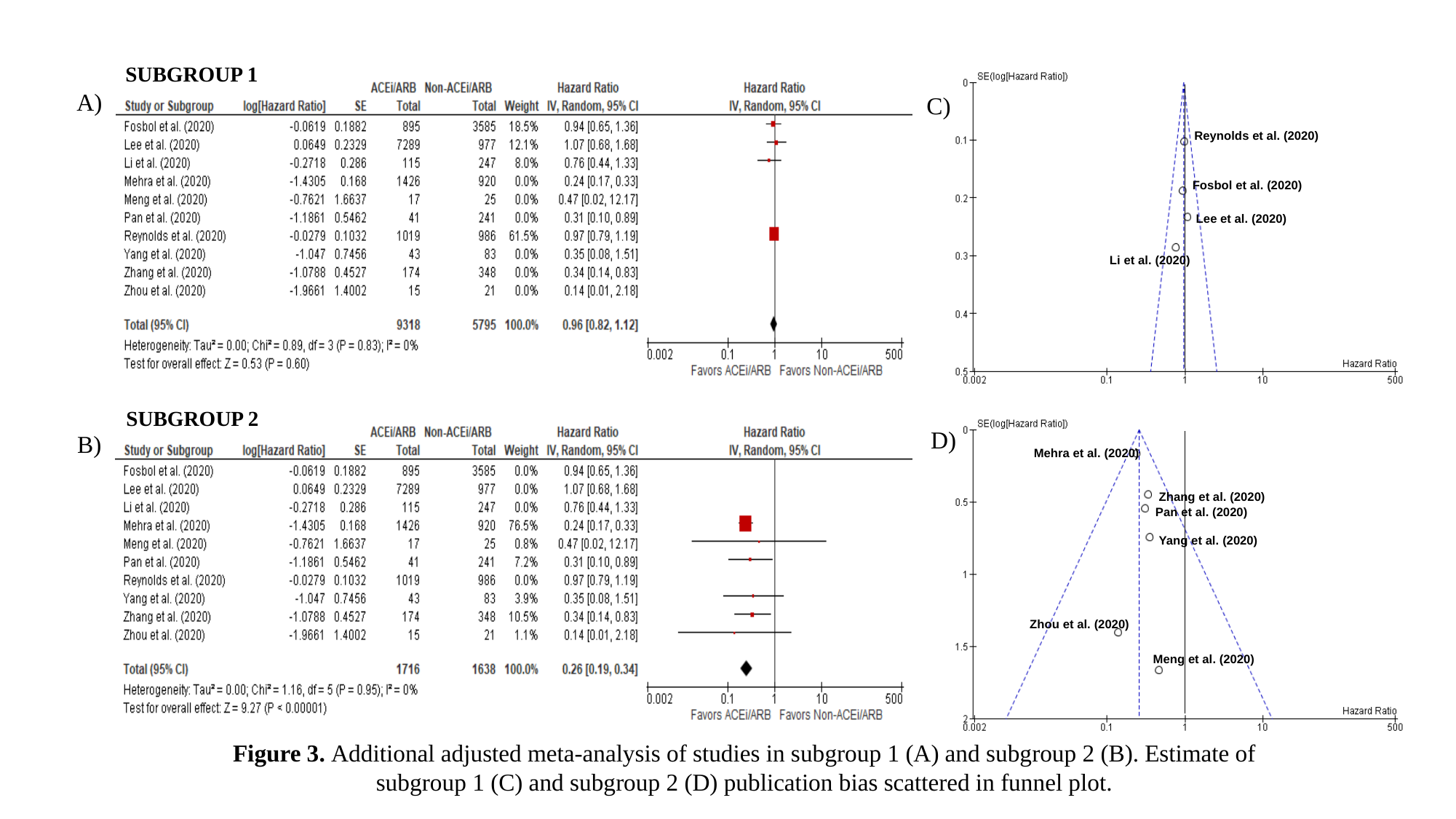

SUBGROUP 1
Reynolds et al. (2020)
Fosbol et al. (2020)
Lee et al. (2020)
Li et al. (2020)
A)
C)
SUBGROUP 2
Mehra et al. (2020)
Zhang et al. (2020)
Pan et al. (2020)
Yang et al. (2020)
Zhou et al. (2020)
Meng et al. (2020)
D)
B)
Figure 3. Additional adjusted meta-analysis of studies in subgroup 1 (A) and subgroup 2 (B). Estimate of subgroup 1 (C) and subgroup 2 (D) publication bias scattered in funnel plot.
